# A ruptured penetrating atherosclerotic ulcer of the ascending aorta: a case report of an endovascular repair with extending the length of the aortic coverage by debranching the innominate artery

**DOI:** 10.1093/ehjcr/ytz043

**Published:** 2019-04-27

**Authors:** Josephine Chenesseau, Pierre-Antoine Barral, Philippe Piquet, Marine Gaudry

**Affiliations:** 1 Department of Vascular Surgery, APHM, Timone Hospital, 264 rue Saint Pierre, Marseille, France; 2 Department of Radiology, APHM, Timone Hospital, 264 rue Saint Pierre, Marseille, France

**Keywords:** Ascending aorta, Acute plaque rupture, Thoracic endovascular aortic repair, Case report

## Abstract

**Background:**

An endovascular approach to the management of a ruptured plaque in the ascending aorta may be an alternative to open surgery in high-risk patients. This option may become inevitable due to the number of elderly patients unfit for open cardiac surgery. There are very few stent grafts able to fit the ascending aorta and in emergency cases, most medical teams have been limited to current thoracic aortic endografts, the shortest of which measure 10 cm.

**Case summary:**

We report a case of an endovascular repair of a ruptured penetrating atherosclerotic ulcer of the ascending aorta. The patient was considered for open cardiac surgery but was evaluated at a high mortality risk based on his age, his medical history, and significant calcifications on his aorta. Our vascular surgical team decided then to perform an endovascular repair with extending the length of the aortic coverage by debranching the innominate artery.

**Discussion:**

Endovascular treatment of an acute ruptured aorta is feasible in high-risk patients with thoracic endovascular stent grafts and coverage of the innominate artery. Endovascular treatment of the ascending aorta is at its infancy and in need of further research. New stent grafts designed for the ascending aorta are in progress and should increase the numbers of interventions in the years to come.

**Learning points**

- An endovascular approach to the management of a ruptured plaque in the ascending aorta may be an alternative to open surgery in high-risk patients.
- There are very few stent grafts able to fit the ascending aorta and in emergency cases, most medical teams have been limited to current thoracic aortic endografts, the shortest ones measure 10 cm.
- We reported a solution to perform an endovascular repair by extending the length of the aortic coverage by debranching the brachiocephalic artery.

## Introduction

An endovascular approach of a ruptured penetrating atherosclerotic ulcer in the ascending aorta may be an alternative to open surgery in high-risk patients. There are very few stent grafts able to fit the ascending aorta and in emergency cases, most medical teams have been limited to current thoracic aortic endografts, the shortest ones measure 10 cm. Here, we describe a case of endovascular repair of the ascending aorta extending the length of the aortic coverage by debranching the innominate artery (IA).

## Timeline

**Table:** 

14 November 2017 09:00 AM	The patient had severe retrosternal chest pain
14 November 2017 03:00 PM	Computed tomography (CT) scan: acute plaque rupture of the ascending aorta with active contrast leakage. The leak is located close to the innominate artery
14 November 2017 06:00 PM	Transferred to intensive care unit of our institution
14 November 2017 07:00 PM	Surgical treatment was carried out.
	First step: innominate artery debranching with a left to right common carotid bypass.
	Second step: thoracic endovascular aortic repair with transapical access
15 November 2017	Sudden right hemiparesis for 48 h followed by complete recovery.
	Normal cerebral CT scan.
04 December 2017	Discharged from the intensive care unit.
14 December 2017	Control CT scan: successful exclusion of the rupture.
15 December 2017	Discharged from our hospital.
26 June 2018	Good clinical evolution: the patient was asymptomatic and doing well without any chest pain and without any neurological sequelae.

## Case presentation

**Figure 1:**
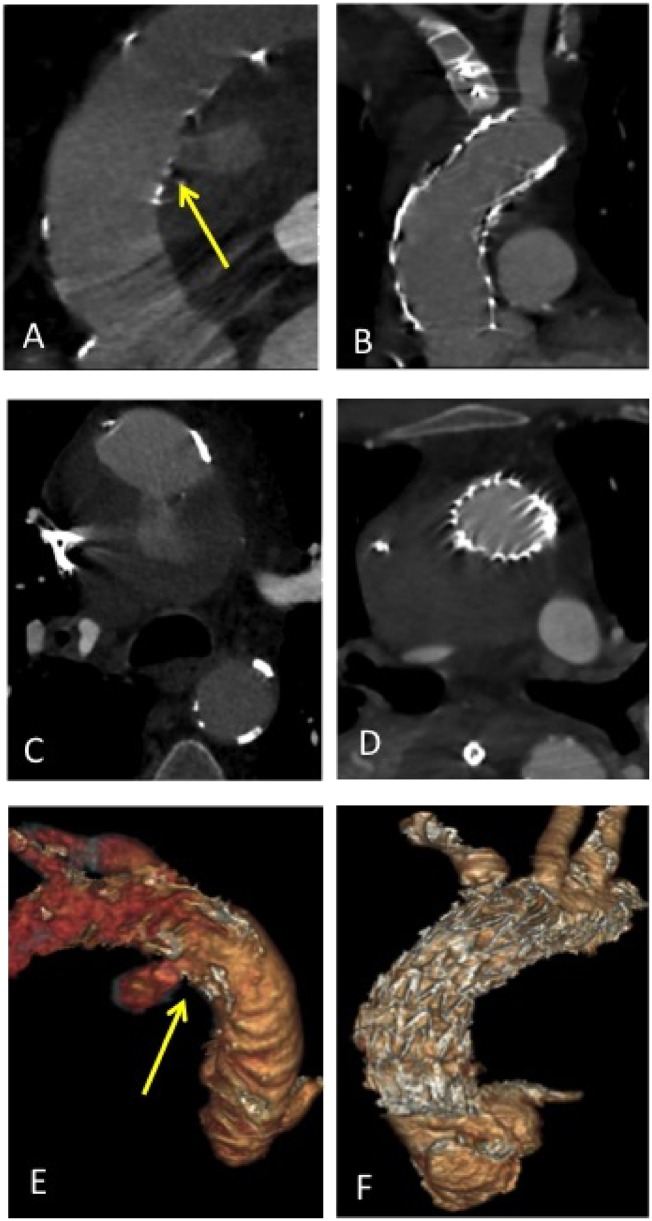
(*A–D*) Initial computed tomography (*A* and *C*) and post-operative implantation (*B* and *D*). (*E* and *F*) Three-dimensional volume rendering reconstruction of initial computed tomography (*E*) and post-operative computed tomography (*F*). (*A*, *C*, and *E*) Local aortic plaque rupture of the ascending aorta with active contrast leakage. The leak is located close to the innominate artery (yellow arrow). (*B* and *D*) Post-operative computed tomography with off the shelf thoracic stent graft Conformable GORE^®^ TAG^®^ Thoracic Endoprothesis (WL Gore & associates, Flagstaff, AZ, USA) with rupture exclusion. (*F*) The shelf thoracic stent graft Conformable GORE^®^ TAG^®^ Thoracic Endoprothesis (WL Gore & associates, Flagstaff, AZ, USA) covering the IA.

A 79-year-old man was admitted to the intensive care unit on November 2017 for a ruptured penetrating atherosclerotic ulcer of the ascending aorta identified on CT scan (*Figure**1*).

He had a significant past medical history: end-stage renal failure undergoing dialysis, and myocardial infarctions with previous stentings (the last infarction dating back 10 months). Earlier in the day, the patient had presented with severe retrosternal chest pain for which a myocardial infarction was initially suspected, but the electrocardiography and the transthoracic echography performed in emergency excluded the diagnosis. A CT scan was realized and showed a local aortic plaque rupture of the ascending aorta with active contrast leakage, the patient was then transferred to our institution.

At admission the patient was haemodynamically stable with good blood pressure, a normal pulmonary auscultation with oxygen saturation at 100%, and no more pain. The patient was considered for open cardiac surgery but was evaluated at a high mortality risk based on his age, his medical history, and significant calcifications on his aorta. Our vascular surgical team decided then to perform an endovascular repair.

In emergency, an off the shelf endovascular stent graft was used. The rupture measured 12 mm and was located at just a few millimetres proximal to the IA. The ascending aorta measured 8 cm from the coronary sinuses to the IA. The largest diameter measured along the length of the ascending aorta was 33 mm (*Figure** 1*). The shortest available off the shelf thoracic stent graft was the 10 cm length Conformable GORE^®^ TAG^®^ Thoracic Endoprothesis (WL Gore & associates, Flagstaff, AZ, USA).

**Figure 2:**
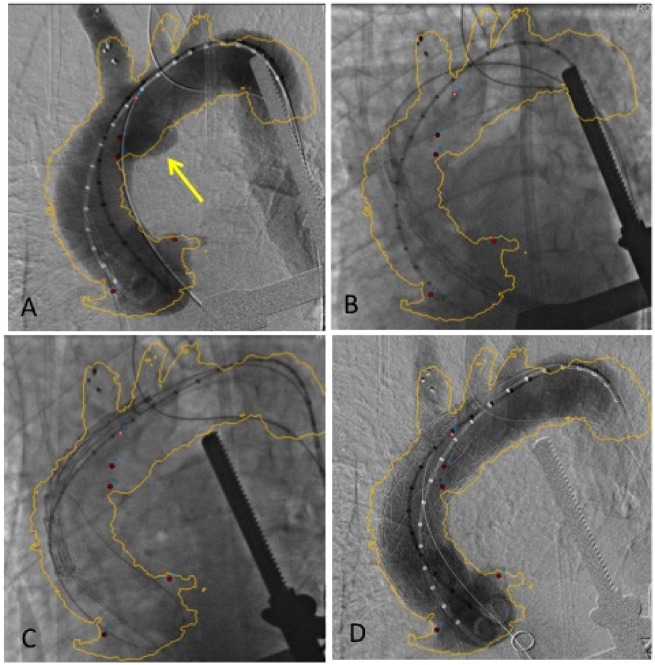
Periprocedural angiography in the hybrid operating room with 3-dimensional image fusion-guided thoracic endovascular aortic repair (see Supplementary material online, *Videos*). Red points were positioned on the coronary ostia, on the ostium of the innominate artery, and on the rupture. (*A*) Angiography before stent graft exclusion showing the aortic plaque rupture (yellow arrow). (*B* and *C*) Stent graft implantation. (*D*) Angiography after stent graft implantation with rupture exclusion.

In a hybrid operative room, first we performed an IA debranching with a left to right common carotid bypass through a cervicotomy using GORETEX vascular graft (8 mm). Via a transfemoral approach, the IA was embolized with a plug emplatzer (16 mm). Then, we considered a femoral approach to place the stent graft, but due to bad femoral access and after determining the working length of the delivery catheter would be insufficient to reach the most proximal landing in the ascending aorta, we decided a transapical approach would be necessary. A 24-Fr introducer sheath was placed through a left ventricular transapical approach by using a mini-thoracotomy. The endograft was deployed under rapid pacing with three-dimensional image fusion-guided thoracic endovascular aortic repair (*Figure**2*). The intra-procedural aortography demonstrated a well-positioned endograft with successful seal of the rupture.

During post-operative period, the patient underwent an ischaemic stroke with sudden right hemiparesis for 48 h followed by complete recovery. The patient was discharged from the intensive care unit at 19 days and from the hospital at 30 days with an antiplatelet therapy (aspirin 75 mg).

**Figure 3:**
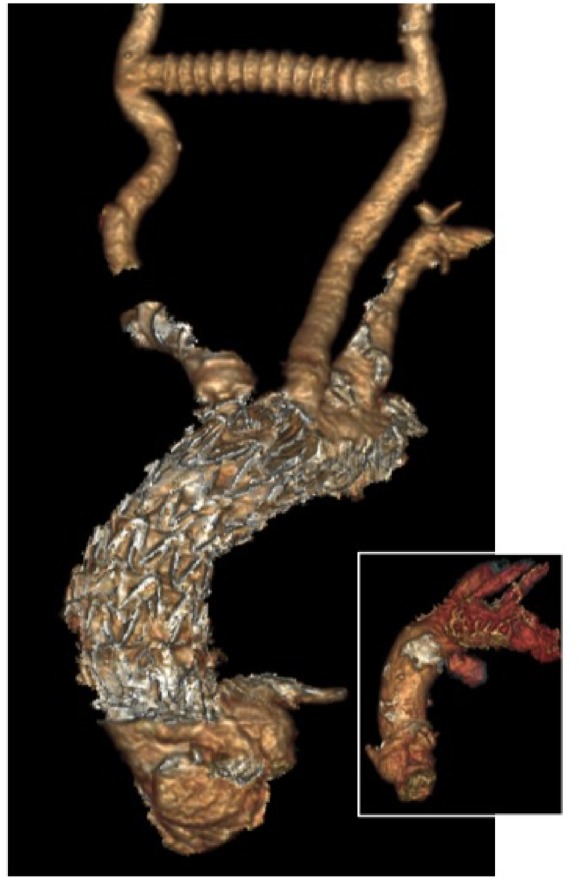
Control computed tomography scan: endovascular stent graft covering the innominate artery in acute aortic plaque rupture.

The post-operative CT scan showed a successful exclusion of the rupture (*Figure**3*). Eight months after surgical treatment, the patient is in an excellent neurological condition without sequelae, and he recovers a quality of life equivalent to the previous state.

## Discussion

The decision to use endovascular repair for treatment of aortic ascending pathologies is not an obvious choice because of its unknown results. More than 50% of these procedures described in the literature were completed for Type A dissections with only three acute ruptured aortas described in a review in 2018.^1^^–^^3^

In our case, regarding the high risk of mortality associated with open surgery and cross-clamping of the aorta (13.30% using the European System for Cardiac Operative Risk Evaluation, EuroSCORE). Our cardiovascular surgical team decided to perform an endovascular repair instead of risking the patient to cardiopulmonary bypass.

There are few stent grafts able to fit the ascending aorta and in emergency cases, most medical teams have been limited to current thoracic aortic endografts.^2^ The shortest measure 10 cm. In our case, we describe a solution to extend the length of the aortic distal neck by debranching the IA to provide adequate coverage over the rupture as well as enough length for placement of the thoracic endoprosthesis. We had to cover the IA because the rupture was located very close (<10 mm) to its ostium.

Due to bad femoral access and the size of the introducer (24 Fr), we decided on a transapical approach.^4^ Deployment of the endograft in the ascending aorta can be challenging and requires precise placement. In our opinion, the procedure is only possible in a hybrid operative room using three-dimensional image superposition and must be performed by a team with experience in the field of endovascular interventions. We believe rapid pacing reduces the risk of graft migration during deployment.^1^

The patient’s post-operative complication is comparable to what is described in the literature.^5^ His 19 day long in-hospital stay in intensive care unit was necessary due to his severe morbidities and the need for invasive ventilation for post-operative pulmonary congestion. The patient was discharge from the hospital after 30 days with a quality of life equivalent to the previous state, an open sternotomy with a cardiopulmonary bypass would have further complicated his recovery with possible additional complications.^1^^,^^4^

Endovascular treatment of the ascending aorta is at its infancy and needs further research. This option will continue to be inevitable due to the number of elderly patients unfit for open cardiac surgery. New stent grafts designed for the ascending aorta are in progress and should increase the numbers of interventions in the years to come.

## Supplementary material

Supplementary material is available at *European Heart Journal - Case Reports* online.

**Slide sets:** A fully edited slide set detailing this case and suitable for local presentation is available online as Supplementary data.

**Consent:** The author/s confirm that written consent for submission and publication of this case report including image(s) and associated text has been obtained from the patient in line with COPE guidance.

**Conflict of interest:** none declared.

## Supplementary Material

ytz043_Supplementary_Video

## References

[1] Muetterties CE, Menon R, Wheatley GH 3rd A systematic review of primary endovascular repair of the ascending aorta. J Vasc Surg 2018;67:332–342. 28844469 10.1016/j.jvs.2017.06.099

[2] Baikoussis NG, Antonopoulos CN, Papakonstantinou NA, Argiriou M, Geroulakos G. Endovascular stent grafting for ascending aorta diseases. J Vasc Surg 2017;66:1587–1601. 28830707 10.1016/j.jvs.2017.07.064

[3] Kratimenos T, Baikoussis NG, Tomais D, Argiriou M. Ascending aorta endovascular repair of a symptomatic penetrating atherosclerotic ulcer with a Custom-Made Endograft. Ann Vasc Surg 2018;47:e1–e4. 10.1016/j.avsg.2017.08.027 28890066

[4] Grieshaber P, Nink N, Roth P, Elzien M, Boning A, Koshty A. Endovascular treatment of the ascending aorta using a combined transapical and transfemoral approach. J Vasc Surg 2018;67:649–655. 29157681 10.1016/j.jvs.2017.10.046

[5] Vallabhajosyula P, Gottret JP, Bavaria JE, Desai ND, Szeto WY. Endovascular repair of the ascending aorta in patients at high risk for open repair. J Thorac Cardiovasc Surg 2015;149:S144–S150. 25218530 10.1016/j.jtcvs.2014.07.063

