# Supplementary material for: A ruptured penetrating atherosclerotic ulcer of the ascending aorta: a case report of an endovascular repair with extending the length of the aortic coverage by debranching the innominate artery
Source: Eur Heart J Case Rep. 2019 Apr 27;3(2):ytz043. doi: 10.1093/ehjcr/ytz043 (PMC6601238; doi:10.1093/ehjcr/ytz043)
Supplement: ytz043_Supplementary_Video [file ytz043_supplementary_video.zip › ytz043_suppl-data/EHJ-CR_Slide_Set-2.pptx]

## Slide 1
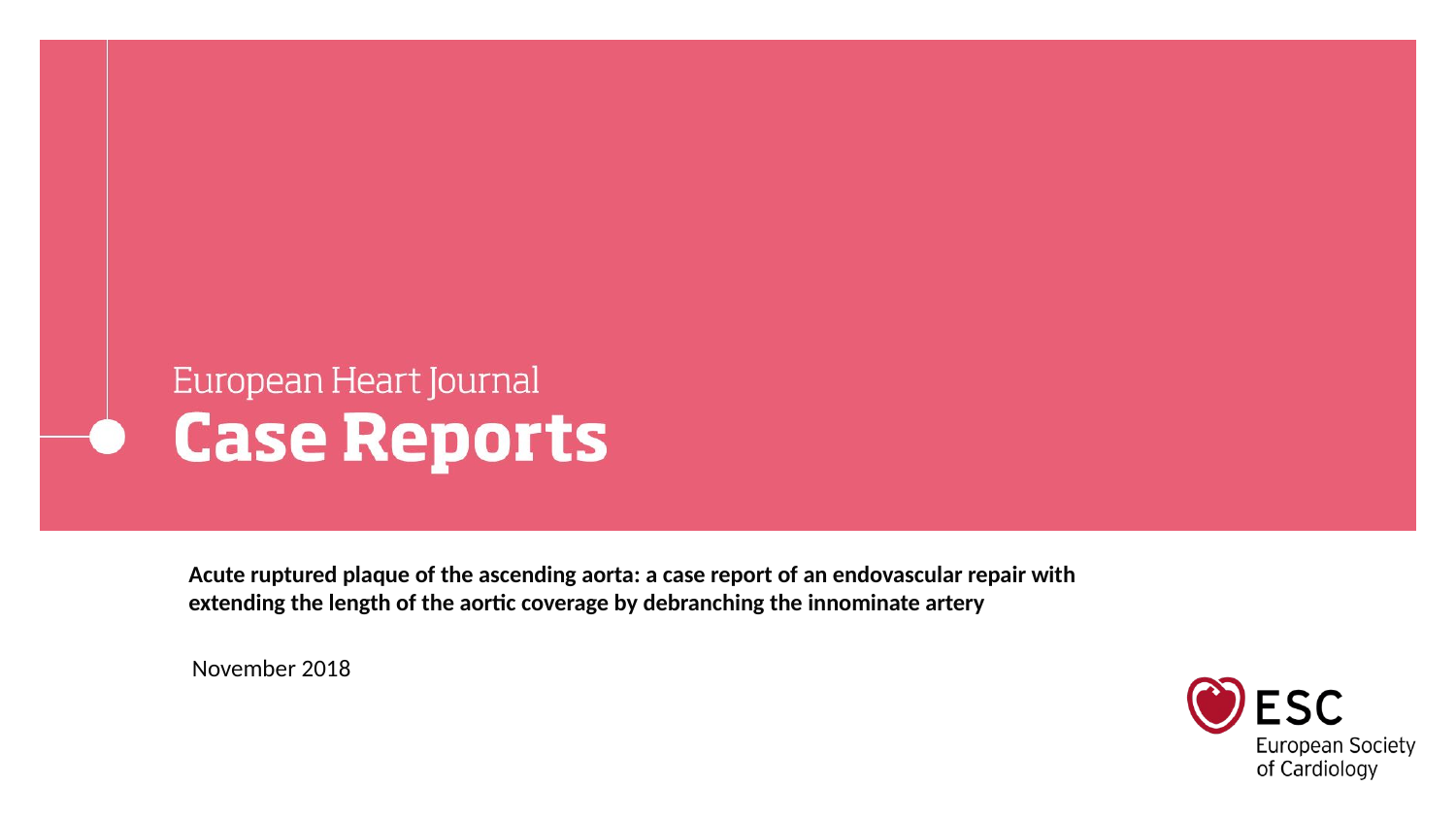

# Acute ruptured plaque of the ascending aorta: a case report of an endovascular repair with extending the length of the aortic coverage by debranching the innominate artery
November 2018

## Slide 2
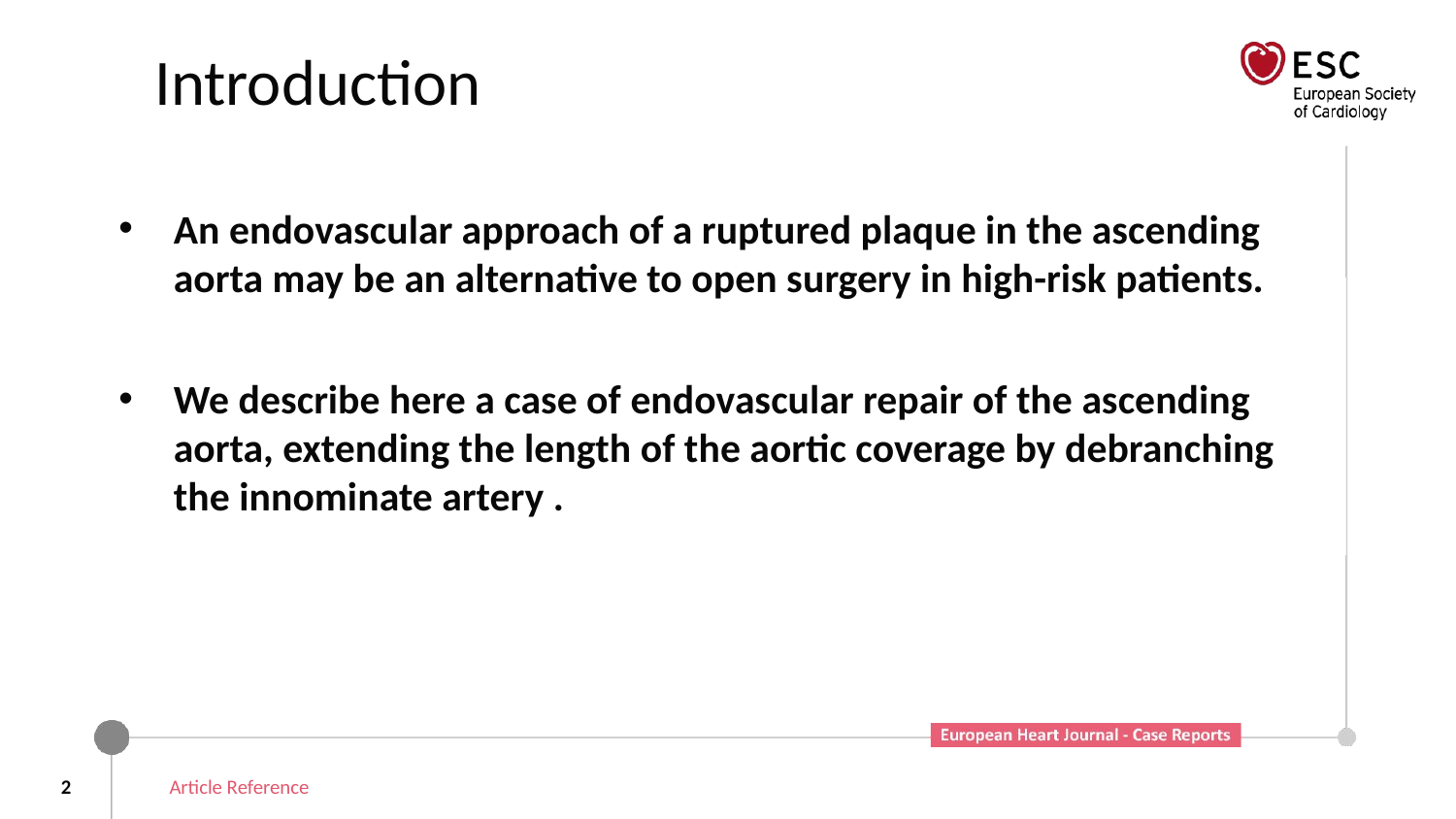

# Introduction
An endovascular approach of a ruptured plaque in the ascending aorta may be an alternative to open surgery in high-risk patients.
We describe here a case of endovascular repair of the ascending aorta, extending the length of the aortic coverage by debranching the innominate artery .
2
Article Reference

## Slide 3
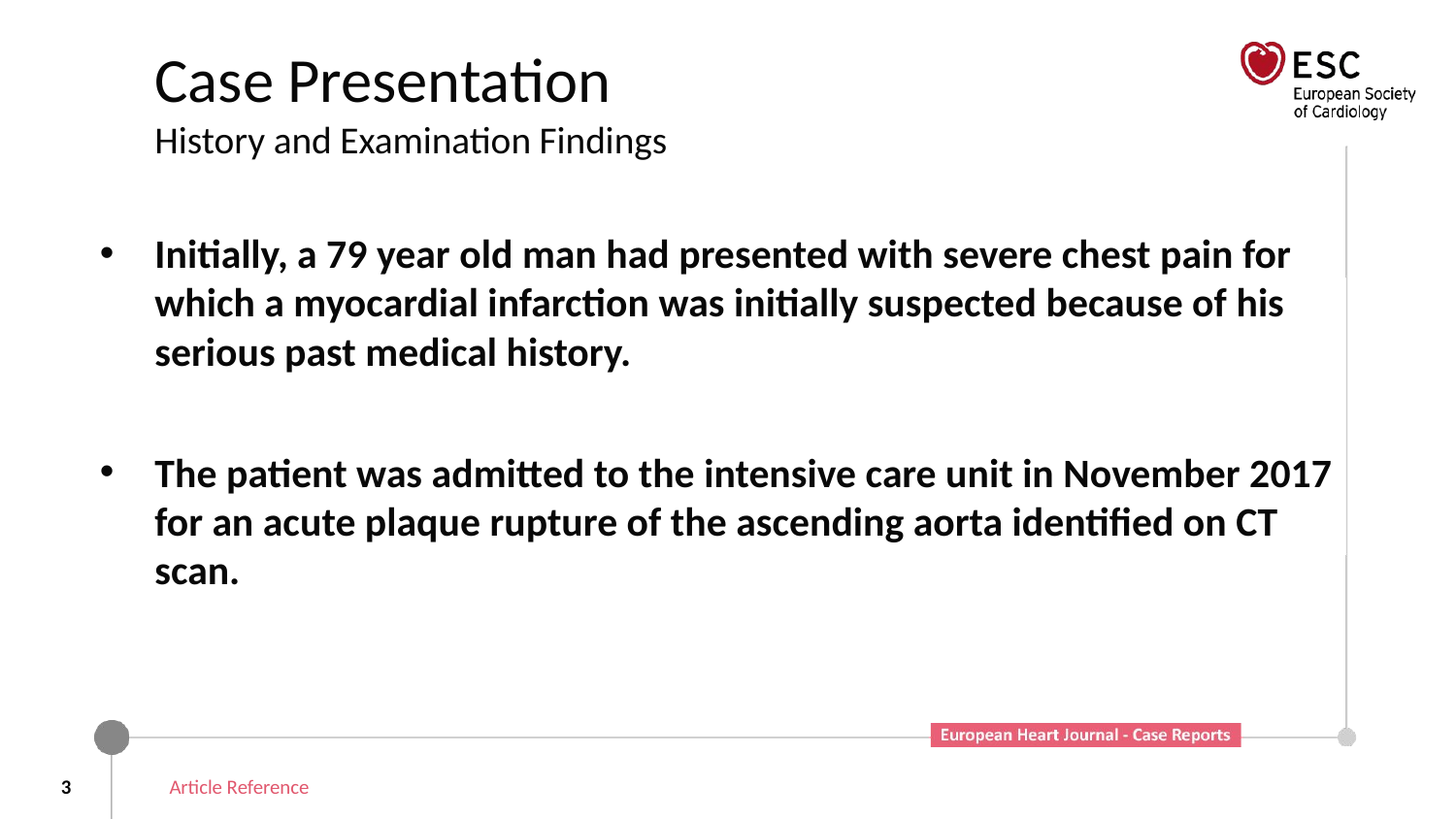

# Case Presentation History and Examination Findings
Initially, a 79 year old man had presented with severe chest pain for which a myocardial infarction was initially suspected because of his serious past medical history.
The patient was admitted to the intensive care unit in November 2017 for an acute plaque rupture of the ascending aorta identified on CT scan.
3
Article Reference

## Slide 4
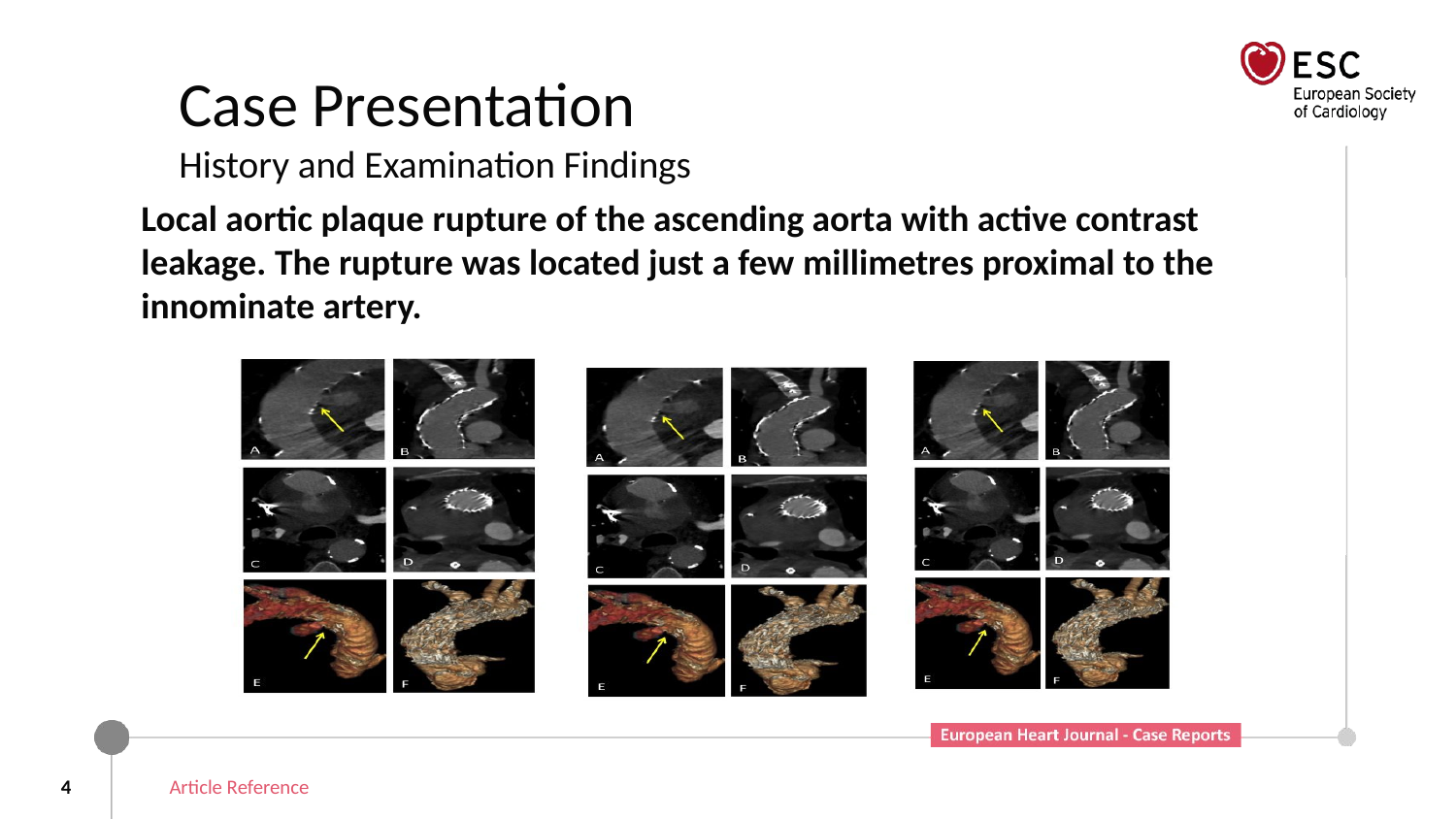

Case Presentation
History and Examination Findings
Local aortic plaque rupture of the ascending aorta with active contrast leakage. The rupture was located just a few millimetres proximal to the innominate artery.
4
Article Reference

## Slide 5
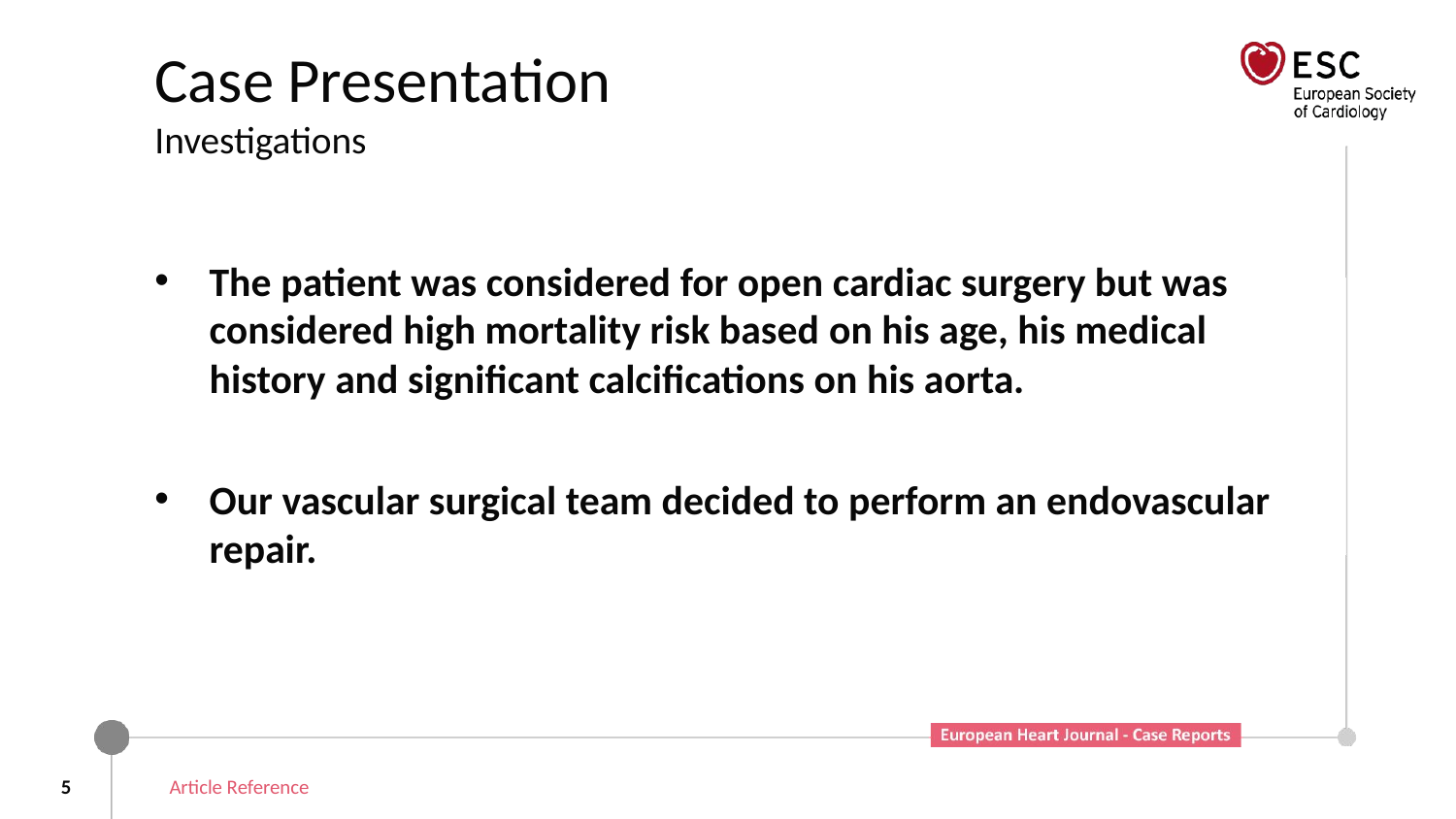

# Case Presentation Investigations
The patient was considered for open cardiac surgery but was considered high mortality risk based on his age, his medical history and significant calcifications on his aorta.
Our vascular surgical team decided to perform an endovascular repair.
5
Article Reference

## Slide 6
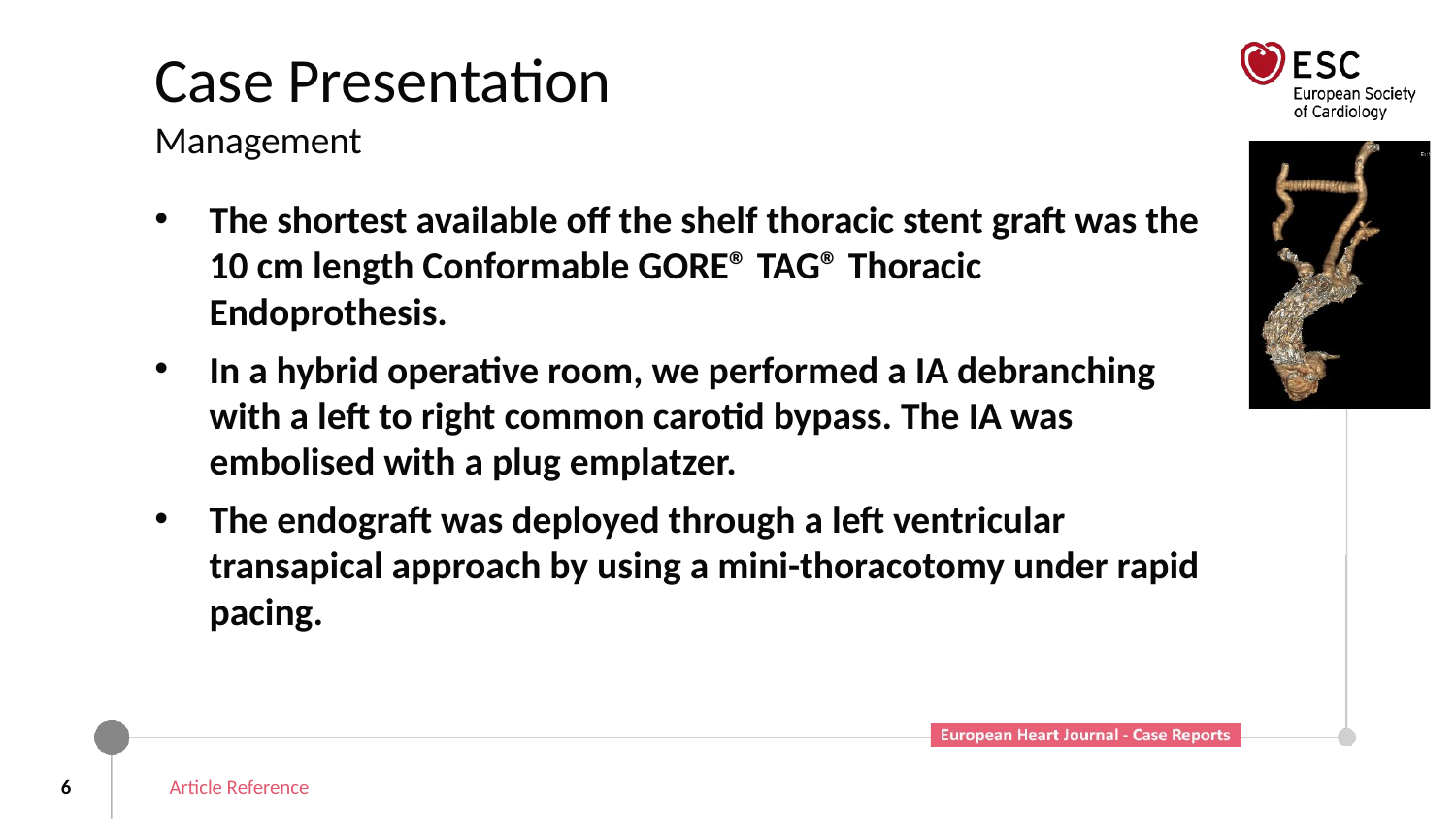

# Case Presentation Management
The shortest available off the shelf thoracic stent graft was the 10 cm length Conformable GORE® TAG® Thoracic Endoprothesis.
In a hybrid operative room, we performed a IA debranching with a left to right common carotid bypass. The IA was embolised with a plug emplatzer.
The endograft was deployed through a left ventricular transapical approach by using a mini-thoracotomy under rapid pacing.
6
Article Reference

## Slide 7
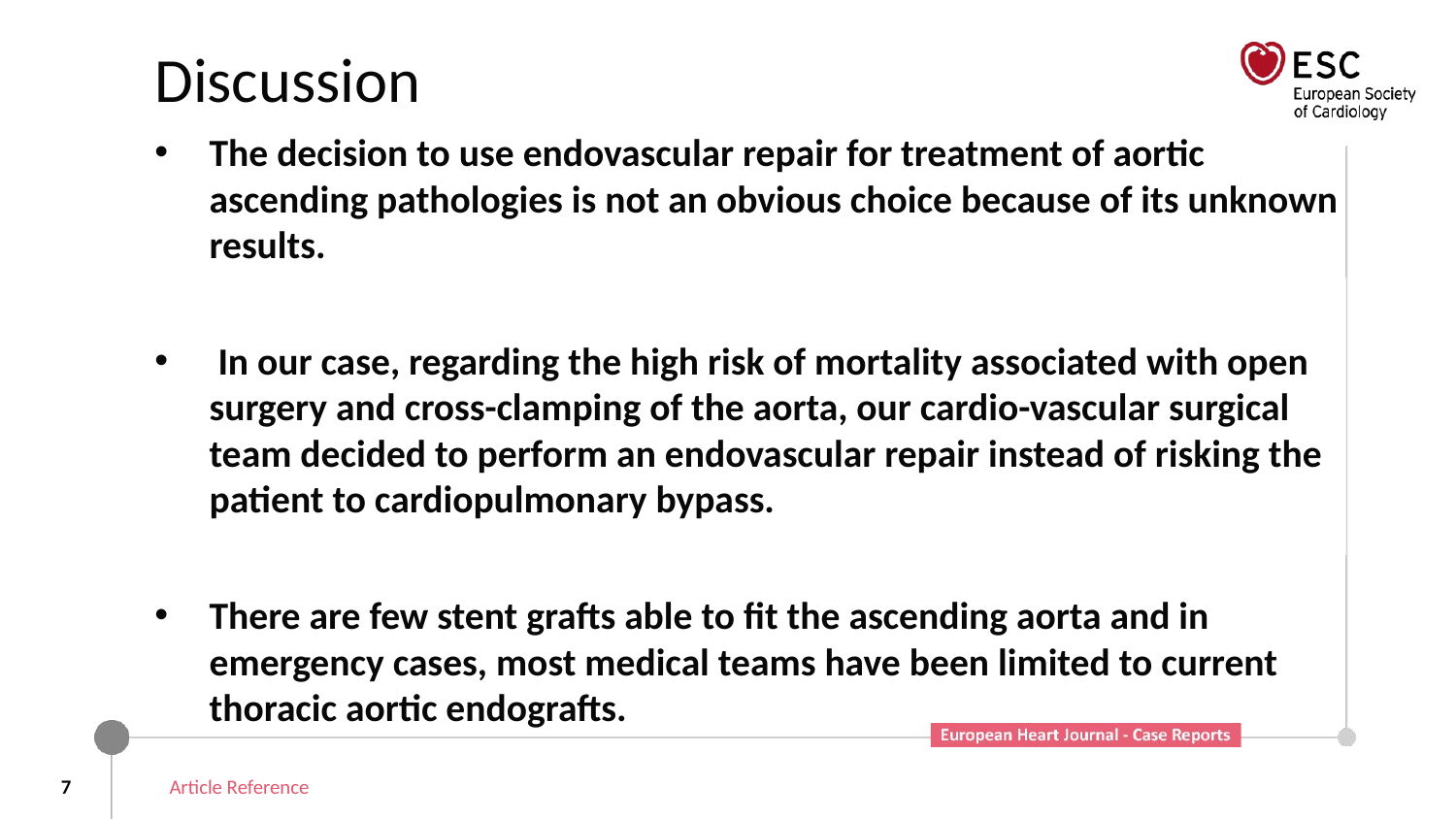

# Discussion
The decision to use endovascular repair for treatment of aortic ascending pathologies is not an obvious choice because of its unknown results.
 In our case, regarding the high risk of mortality associated with open surgery and cross-clamping of the aorta, our cardio-vascular surgical team decided to perform an endovascular repair instead of risking the patient to cardiopulmonary bypass.
There are few stent grafts able to fit the ascending aorta and in emergency cases, most medical teams have been limited to current thoracic aortic endografts.
7
Article Reference

## Slide 8
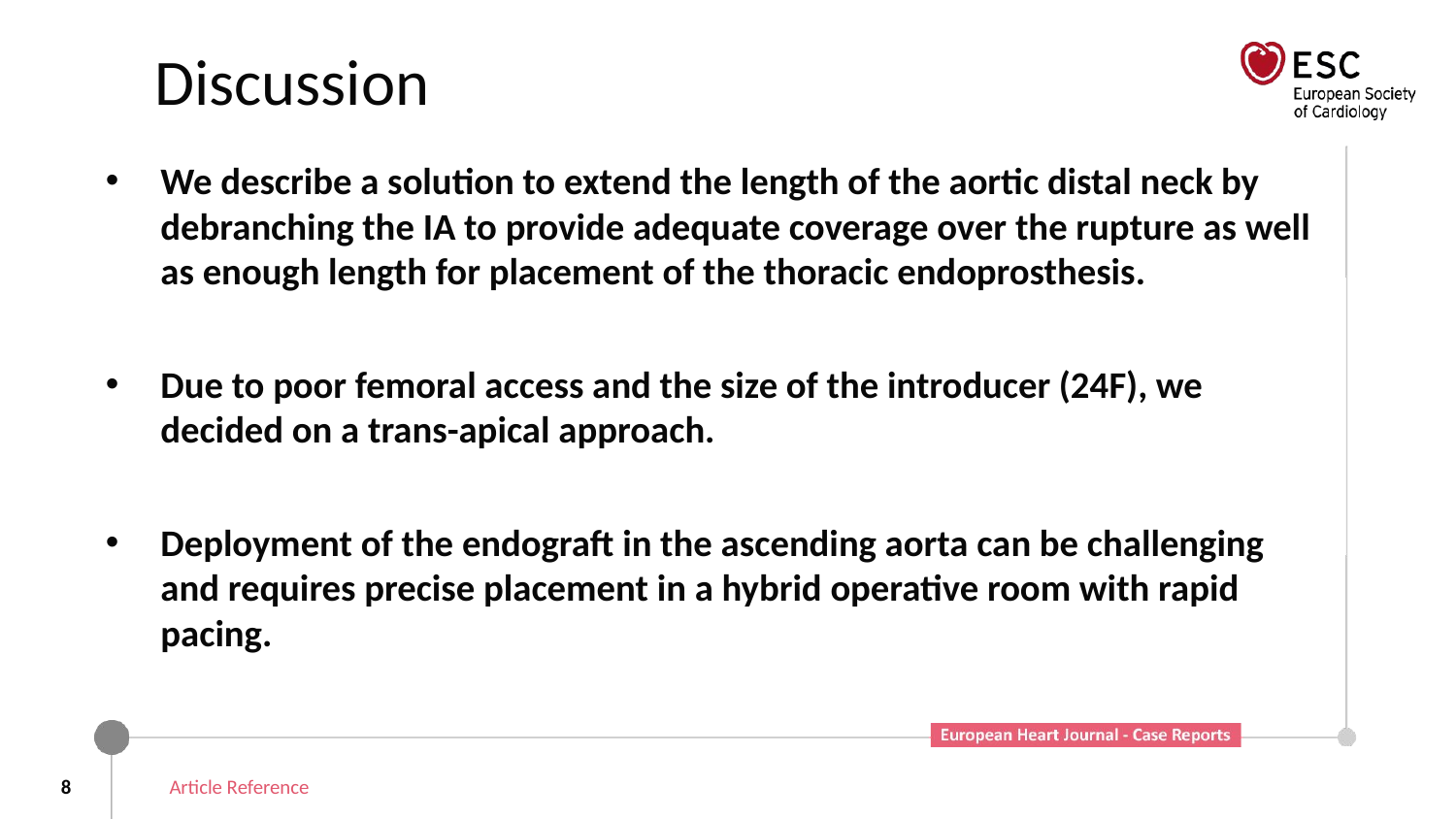

# Discussion
We describe a solution to extend the length of the aortic distal neck by debranching the IA to provide adequate coverage over the rupture as well as enough length for placement of the thoracic endoprosthesis.
Due to poor femoral access and the size of the introducer (24F), we decided on a trans-apical approach.
Deployment of the endograft in the ascending aorta can be challenging and requires precise placement in a hybrid operative room with rapid pacing.
8
Article Reference

## Slide 9
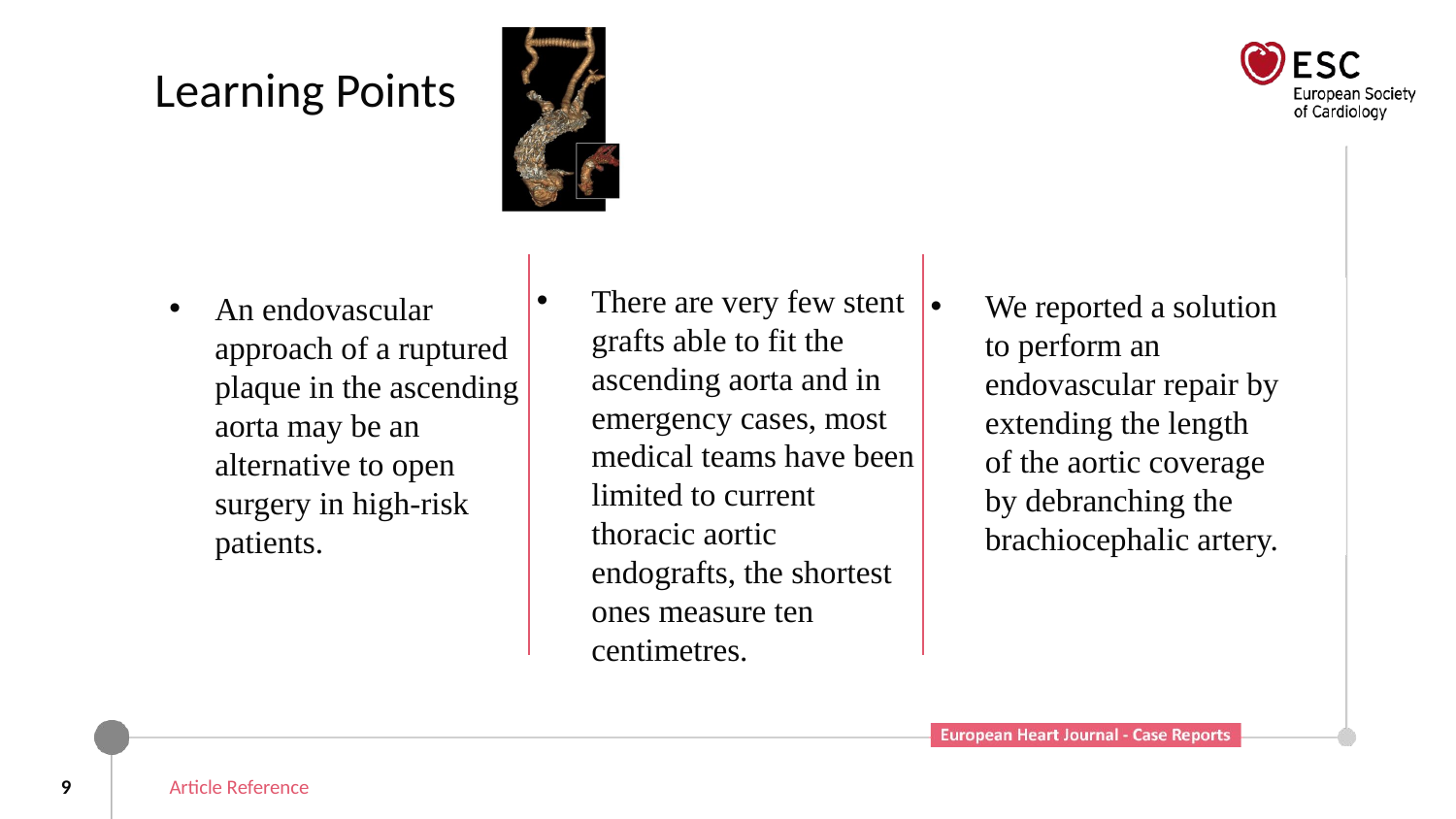

# Learning Points
There are very few stent grafts able to fit the ascending aorta and in emergency cases, most medical teams have been limited to current thoracic aortic endografts, the shortest ones measure ten centimetres.
We reported a solution to perform an endovascular repair by extending the length of the aortic coverage by debranching the brachiocephalic artery.
An endovascular approach of a ruptured plaque in the ascending aorta may be an alternative to open surgery in high-risk patients.
9
Article Reference

## Slide 10
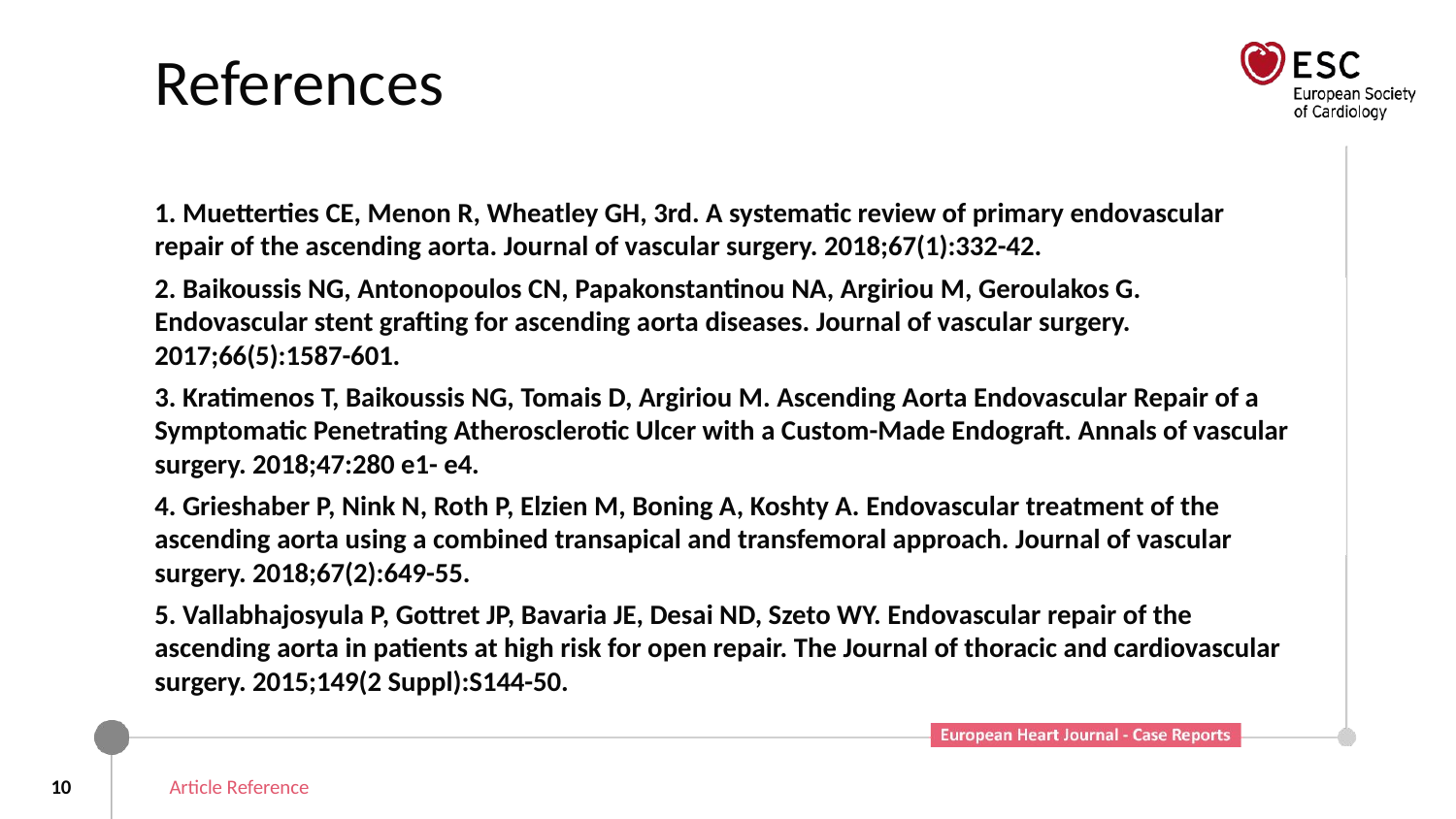

# References
1. Muetterties CE, Menon R, Wheatley GH, 3rd. A systematic review of primary endovascular repair of the ascending aorta. Journal of vascular surgery. 2018;67(1):332-42.
2. Baikoussis NG, Antonopoulos CN, Papakonstantinou NA, Argiriou M, Geroulakos G. Endovascular stent grafting for ascending aorta diseases. Journal of vascular surgery. 2017;66(5):1587-601.
3. Kratimenos T, Baikoussis NG, Tomais D, Argiriou M. Ascending Aorta Endovascular Repair of a Symptomatic Penetrating Atherosclerotic Ulcer with a Custom-Made Endograft. Annals of vascular surgery. 2018;47:280 e1- e4.
4. Grieshaber P, Nink N, Roth P, Elzien M, Boning A, Koshty A. Endovascular treatment of the ascending aorta using a combined transapical and transfemoral approach. Journal of vascular surgery. 2018;67(2):649-55.
5. Vallabhajosyula P, Gottret JP, Bavaria JE, Desai ND, Szeto WY. Endovascular repair of the ascending aorta in patients at high risk for open repair. The Journal of thoracic and cardiovascular surgery. 2015;149(2 Suppl):S144-50.
10
Article Reference
